# Novel Target Opportunities in Non-Metastatic Castrate Resistant Prostate Cancer

**DOI:** 10.3390/cancers13102426

**Published:** 2021-05-17

**Authors:** Stephanie Gleicher, Baylee A. Porter, Disharee Nath, Guanqun Li, Rakesh Khanna, Hanan Goldberg, Marcin Kortylewski, Gennady Bratslavsky, Leszek Kotula

**Affiliations:** 1 Department of Urology, SUNY Upstate Medical University, 750 East Adams Str., Syracuse, NY 13010, USA; gleiches@upstate.edu (S.G.); porterba@upstate.edu (B.A.P.); disharee.nath@gmail.com (D.N.); lig@upstate.edu (G.L.); rvkhann@gmail.com (R.K.); goldberh@upstate.edu (H.G.); bratslag@upstate.edu (G.B.); 2 Department of Biochemistry and Molecular Biology, SUNY Upstate Medical University, 750 East Adams Str., Syracuse, NY 13010, USA; 3 Beckman Research Institute, City of Hope, 1500 E. Duarte Road, Beckman Center Room 3111, Duarte, CA 91010, USA; mkortylewski@coh.org

**Keywords:** prostate cancer, castrate resistance, non-metastatic CRPC, clinical trial, epithelial mesenchymal transition, STAT3

## Abstract

**Simple Summary:**

Following local treatment of prostate cancer by surgical removal or radiation, biochemical recurrence may occur and progress to castration resistance (CR) following androgen deprivation therapy (ADT). If disease persists, men develop metastatic disease (mCRPC) which leads to death. Prior to mCRPC, a non-metastatic state exists (nmCRPC) characterized by a rise in PSA and lack of detectable metastases. Here, we review potential therapeutic strategies to interfere with the transition before the cancer becomes deadly.

**Abstract:**

Nearly one third of men will incur biochemical recurrence after treatment for localized prostate cancer. Androgen deprivation therapy (ADT) is the therapeutic mainstay; however, some patients will transition to a castrate resistant state (castrate resistant prostate cancer, CRPC). Subjects with CRPC may develop symptomatic metastatic disease (mCRPC) and incur mortality several years later. Prior to metastatic disease, however, men acquire non-metastatic CRPC (nmCRPC) which lends the unique opportunity for intervention to delay disease progression and symptoms. This review addresses current therapies for nmCRPC, as well as novel therapeutics and pathway strategies targeting men with nmCRPC.

## 1. Introduction

In the United States, prostate cancer is the most common cancer among men with approximately 175,000 new diagnoses per year [1]. Among men who undergo therapy for localized disease, nearly one third will develop biochemical recurrence as assessed by a rise in prostate specific antigen (PSA) [2]. Given the androgen sensitive nature of prostate cancer, men are generally started on androgen deprivation therapy (ADT) after they recur [3]. ADT includes luteinizing hormone releasing hormone (LHRH) agonists (i.e., leuprolide, goserelin, histrelin), LHRH antagonists (i.e., degarelix) [4], or the very recently US Food and Drug Administration (FDA) approved oral LHRH antagonist, relugolix [5]. Despite suppression of androgen signals, many hormone sensitive prostate cancer patients will eventually transform into hormone refractory, or castrate resistant prostate cancer (CRPC), which carries a poor prognosis and has high rates of metastatic disease (mCRPC). mCRPC is ultimately what causes symptoms and death among prostate cancer subjects with a median survival time around 3–4 years [6,7]. Studies have shown that nearly 50% of men with nonmetastatic CRPC (nmCRPC) will develop metastases after two years [8]. It is important to note that new imaging modalities including PSMA-PET, NaF PET, and 11C-choline PET/CT will likely change the landscape of nmCRPC with earlier identification of measurable metastatic disease [9]. However, current disease progression from nmCRCP to mCRPC typically occurs after 48 months. Given this knowledge, treatment of nmCRPC subjects offers a unique opportunity to delay progression to mCRPC. There has been a surge in research in this domain with three new anti-androgen agents achieving FDA approval in 2018 and 2019 for combination therapy with ADT in the setting of nmCRPC. The goal of this review is to discuss current anti-androgen treatment options for nmCRPC, as well as innovative non-androgen based therapeutic targets that have been explored. Lastly, we will discuss a novel pathway, epithelial mesenchymal transition (EMT) process, that may have utility in subjects with nmCRPC to delay disease progression. 

## 2. Current Treatment Paradigm for nmCRPC

**Figure 1:**
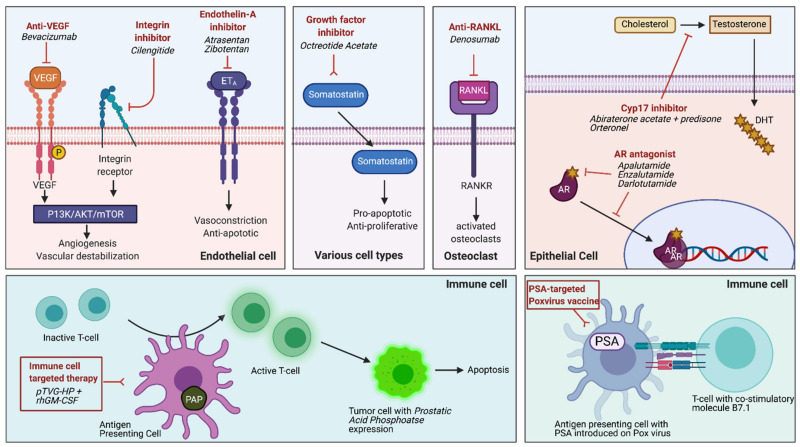
Major pathways targeted in clinical trials of nmCRPC. These include panels from left to right: angiogenesis and vascular destabilization (Anti-VEGF and Endothelin inhibitor); cell matrix adhesion/EMT (Integrin inhibitor); growth hormone pathway (Somatostatin); androgen pathway (AR inhibitors); bone metastases inhibitor (anti-RANKL); immune cell therapy (rhGM-CSF); and PSA based vaccines (PSA-Pox virus). See Table 1 for clinical trials information related to these targets.

In 2018 and 2019, three second generation anti-androgen therapeutics were approved by the FDA for combination therapy with ADT in the setting of nmCRPC with PSA doubling time (PSADT) < 10 months: apalutamide, enzalutamide, and darolutamide [4,10,11,12]. These small molecule compounds act by three different mechanisms: inhibiting androgen binding to the androgen receptor, inhibiting androgen receptors from entering the nucleus, and inhibiting androgen receptor binding to DNA (Figure 1) [10,11,12]. They also bind to the androgen receptor with a higher affinity than the first-generation anti-androgens (i.e., flutamide, bicalutamide, nilutamide) which solely prevent androgen receptor translocation to the nucleus [13,14]. 

Apalutamide, a nonsteroidal antiandrogen, binds directly to the ligand-binding domain of the androgen receptor with a 7- to 10-fold higher affinity versus first-generation agents [10,13]. It is a selective and competitive androgen receptor inhibitor [14]. The Selective Prostate Androgen Receptor Targeting with ARN-509 (SPARTAN) trial was a randomized controlled trial comparing apalutamide with placebo in patients who were at high risk of developing metastasis as defined by a PSA doubling time of less than 10 months. This trial showed that when combined with ADT, the addition of Apalutamide resulted in a metastasis free survival (MFS) of 40.5 months compared to 16.2 months with the combination of ADT and placebo [10]. Of note, the apalutamide group did have a higher incidence of rash (23.8% versus 5.5%), hypothyroidism (8.1% versus 2%) and fracture (11.7% versus 6.5%) [10]. This trial was the basis for the FDA approval of apalutamide as a treatment in nmCRPC (Table 1). 

The PROSPER trial was a large, international, randomized controlled trial comparing the addition of enzalutamide or placebo to ADT. Enzalutamide is an androgen receptor antagonist that also binds with higher affinity than first-generation anti-androgens [14]. Enzalutamide was also approved for use in CRPC per the results from the PREVAIL and AFFIRM trials [6,15]. Eligible patients for the PROSPER trial had a PSA doubling time of less than or equal to 10 months and PSA ≥ 2 ng/mL at screening [11]. Enzalutamide was found to have a MFS of 36.6 months compared to 14.7 months in the placebo group [11]. Enzalutamide also prolonged the use of antineoplastic therapy. Of note, 31% of subjects in the enzalutamide arm had grade 3 or higher adverse events versus 23% in the placebo group [11]. As a result of the PROSPER trial, the FDA approved enzalutamide in the nmCRPC setting. 

The ARAMIS multinational, randomized controlled trial compared Darolutamide with ADT to a placebo with ADT [12]. Darolutamide is also an androgen receptor antagonist that has been found to be more efficacious than apalutamide and enzalutamide. Interestingly, Darolutamide is able to bind to the androgen receptor despite various mutations that impact the efficacy of other second generation anti-androgens (typically converting them from antagonist to agonist) [14]. The results of the ARAMIS trial showed that Darolutamide improved MFS (40.4 months versus 18.4 months) with no significant difference in side effects [12]. In 2019, Darolutamide was also granted FDA approval for treatment of patients with nmCRPC [12].

**Table 1:** Current trials targeting nmCRPC clinical space.

Trial Identifier	Trial Name/Details	Name	Drug Target	Sample Size (*n*)	Conclusions/Metastasis Free Survival (MFS)
NCT01946204	Selective Prostate Androgen Receptor Targeting with ARN-509 (SPARTAN), Phase 3	Apalutamide	AR antagonist	*n* = 806 ADT/ apalutamide *n* = 401 ADT/placebo	40.5 months versus 16.2 months with placebo (HR 0.28, 95% CI 0.23–0.35, *p* < 0.001) [10]
NCT02003924	PROSPER, Phase 3	Enzalutamide	AR antagonist	*n* = 933 ADT/ enzalutamide *n* = 468 ADT/placebo	36.6 months versus 14.7 months with placebo (HR 0.29, 95% CI 0.24–0.35, *p* < 0.001) [11]
NCT02200614	ARAMIS, Phase 3	Darolutamide	AR antagonist	*n* = 955 ADT/ darolutamide *n* = 554 ADT/placebo	40.4 months versus 18.4 months with placebo (HR 0.41, 95% CI 0.34–0.50, *p* < 0.001) [12]
NCT01314118	IMAAGEN trial Phase 2	Abiraterone acetate + prednisone	Cytochrome C17 enzyme (CYP17), androgen synthesis	*n* = 131 ADT/ abiraterone and prednisone	Median PSA progression at 28.7 months, radiographic progression not reached (95% CI 21.2–38.2) [16]
NCT00121238	Phase 2	Cilengitide	selective antagonist of α_v_ β_3_ and α_v_ β_5_ integrins	*n* = 16 cilengitide	No detectable clinical activity [17]
NCT00036556	Atrasentan, Phase 3	Atrasentan	selective endothelin -A receptor antagonist	*n* = 467 Atrasenten *n* = 474 placebo	No significant delay in time to disease progression, but did show a prolongation of TTP among patients outside the US only [18]
NCT00626548	ENTHUSE M0, Phase 3	Zibotentan	ET_A_ receptor antagonist	*n* = 705 Zibotentan *n* = 716 placebo	No difference in overall survival or progression free survival resulting in trial termination [19]
NCT00510224	Sandostatin, Phase 2	Octreotide Acetate	Insulin-like growth factor (IGF) signaling pathway inhibition, somatostatin analogue that inhibits growth hormone release from the pituitary	*n* = 13 Octreotide	No decline in PSA levels despite three cycles of treatment and a decline in IGF levels [20]
NCT00286091	Phase 3	Denosumab	anti-RANKL monoclonal antibody	*n* = 716 Denosumab *n* = 716 placebo	29.5 months versus 25.2 months with placebo to delay *bone* MFS specifically (HR 0.85, 95% CI 0.73–0.98, *p* = 0.028) [21]
NCT01046916	TAK-700, Phase 2	Orteronel (TAK-700)	CYP17A selective inhibitor	*n* = 39 Orteronel	Median time to PSA progression and metastases to be 14 months and 25 months [22]
NCT00849121	DNA vaccine, Phase 2	DNA Vaccine: pTVG-HP with rhGM-CSF	Sipuleucel-T +/− DNA-based vaccine booster (pTVG-HP) for prostatic acid phosphatase (PAP)	*n* = 9 pTVG-HP *n* = 9 No booster	Repetitive immunization with pTVG-HP maintained antigen-specific T-cells that target prostate cells [23]
N/A	Randomized control trial	Poxvirus-based PSA vaccine	Vaccine with transgenes for PSA and human t-cell costimulatory molecule B7.1; priming vaccine followed by monthly boosts with GM-CSF	*n* = 21 Vaccine *n* = 21 Nilutamide Crossover design with PSA progression	Noted trend toward survival benefit for patients randomized to vaccine arm (median 5.1 years in vaccine vs. 3.4 years in ADT, *p* = 0.13) [24]
NCT01656304	Pilot phase 2 trial	Bevacizumab	Humanized monoclonal antibody against vascular endothelial growth factor (VEGF)	*n* = 15 Bevacizumab	No benefit noted [25]

## 3. Targets beyond Androgen Receptor for the nmCRPC Therapy

Biologic conversion to CRPC is predominantly driven by high AR expression and transcriptional activity [26,27,28]. There are several mechanisms related to AR signaling: AR overexpression from reactivated AR or AR gene amplification, activating mutations, ligand-independent AR splice variants (most notably ARV7, which is a clinical marker of disease progression), AR bypass mechanism from over-activated glucocorticoid receptor expression, and AR low or negative prostate cancer, which is characterized by specific phenotype of neuroendocrine prostate cancer (NEPC) [29,30]. The latter variant is particularly aggressive with median survival of seven months and therapy is limited to platinum-based chemotherapy [31]. Evidence suggests that the number of NEPC will increase due to drug therapy driven resistance or t-NEPC [29]. Given the hormonal nature of CRPC and tendency towards AR resistance, therapeutics that do not target AR have been explored for subjects with nmCRPC. Studies have shown mixed results. This section provides an overview of various non-androgen receptor-based treatments in the setting of nmCRPC. 

Abiraterone acetate is an irreversible CYP17 inhibitor targeting androgen biosynthesis in the testicles, adrenal glands, and prostate cancer tumor cells. The IMAAGEN trial was a phase II, multicenter study that evaluated PSA responses to abiraterone acetate in 131 nmCRPC patients with a PSA higher than 10 ng/mL or a PSADT lower than 10 months (NCT01314118) [16]. The primary endpoint of the study was PSA response at 6 months. The results demonstrated that 87% of patients exhibited a PSA decline of more than 50%. A decline in PSA of over 90% was noted in 60% of subjects [16]. The median time to PSA progression and to radiographic progression was 28.7 months and not reached, respectively [16]. The toxicity profile of abiraterone was similar to that reported in phase III trials assessing its role in mCRPC patients. 

Another small molecule inhibitor of androgen production, orteronel (TAK-700), targeting CYP17A1, was tested in a Phase 2 trial [22]. Data revealed median time to PSA progression and metastases to be 14 months and 25 months, respectively (NCT01046916) [22]. Unfortunately, Phase 3 trials exploring orteronel in mCRPC and hormone sensitive metastatic prostate cancer have not shown a survival benefit (NCT01193244). There are ongoing trials investigating the role of orteronel in high risk localized prostate cancer (NCT01546987) [32,33].

Integrins, a family of transmembrane receptors, have been shown to mediate invasion and angiogenesis in prostate cancer bone metastases. A Phase 2 study investigating the effects of cilengitide, a selective antagonist of α_v_ β_3_ and α_v_ β_5_ integrins, in nmCRPC was completed in 2015 [17]. While cilengitide was well tolerated, it had no detectable clinical activity [17].

Endothelin-1 (ET-1) and the ET_A_ receptor have been implicated in prostate cancer progression. Atrasentan is a selective endothelin-A receptor antagonist. The atrasentan Phase 3 Study Group explored the use of atrasentan in nmCRPC in a randomized placebo-controlled trial. While atrasentan lengthened PSADT and slowed increase in bone alkaline phosphatase levels, this study did not show a significant delay in time to disease progression. However, geographical differences in median time to progression (TTP) were noted: atrasentan did show a prolongation of TTP among patients outside the US, whereas it did not delay TTP among US patients [18]. In another study, the ET_A_ receptor antagonist zibotentan was compared to placebo in patients with nmCRPC [19]. At interim analysis, the zibotentan and placebo groups did not differ in overall survival or progression-free survival resulting in trial termination. The authors concluded that zibotentan was no longer under investigation as a potential treatment for prostate cancer [19]. 

Insulin-like growth factor (IGF) is an endocrine hormone that promotes anabolic activity after signals from growth hormone and has been implicated in the growth of prostate cancer. A Phase 2 trial evaluated the effect of octreotide, a somatostatin analogue that inhibits growth hormone release from the pituitary in men with nmCRPC [20]. The trial was stopped early after a pre-planned interim analysis showed no decline in PSA levels despite three cycles of treatment and a decline in IGF levels [20]. 

Bevacizumab (Avastin), a humanized monoclonal antibody against vascular endothelial growth factor A (VEGF-A), a potent proangiogenic and immunosuppressive mediator, was also trialed in nmCRPC patients (NCT01656304) [25]. Fifteen subjects received treatment every 14 days until PSA progression. Median time to PSA progression and new metastases was noted to be 2.8 months and 7.9 months, respectively. This treatment was deemed ineffective [25]. 

Denosumab is a human RANKL-specific monoclonal antibody that is approved for the prevention of skeletal-related events. A randomized Phase 3 trial was conducted in men with nmCRPC, evaluating the effects of denosumab on bone-metastasis-free survival [21]. Compared to placebo, denosumab significantly increased bone-metastasis-free survival by a median of 4.2 months [21]. However, denosumab was associated with a higher incidence of osteonecrosis of the jaw and hypocalcemia [21]. The FDA denied an expanded indication for denosumab for the prevention of bone metastasis.

Despite the limited efficacy of immune checkpoint blocking antibodies in prostate cancer, several studies suggested the potential of immunization-based strategies in nmCRPC patients. A Phase 2 trial explored the use of a DNA-based vaccine (pTVG-HP) in conjunction with sipuleucel-T, an immunologic agent that stimulates T cells via antigen presenting cells (APCs), to target prostatic acid phosphatase (PAP), a protein specifically produced by the prostate gland [34]. After administration of sipuleucel-T, some subjects received a booster of pTVG-HP with granulocyte-macrophage colony-stimulating factor (GM-CSF) to maintain a PAP-specific T cell response [23,34]. This pilot study suggests that repetitive immunization with the DNA-vaccine maintained an antigen-specific T-cell response after therapy and in a safe manner (NCT00849121) [23,34]. 

Use of a poxvirus-based PSA vaccine (PSA-TRICOM) in conjunction with ADT (nilutamide) in nmCRPC was explored previously [24,35]. The study included 42 subjects who were randomized to receive either the vaccine or ADT. Upon PSA progression without evidence of metastatic disease on imaging, patients could cross-over to receive both therapies. Time to PSA progression was 7.6 months with nilutamide versus 9.9 months with vaccine first [35]. At 6 years, a trend was noted toward survival benefit for patients randomized to the vaccine arm [24]. 

## 4. Novel Epithelial Mesenchymal Transition (EMT) Process to Delay nmCRPC

Novel targeting of the dysregulated epithelial mesenchymal transition (EMT) process may provide opportunity to delay nmCRPC disease progression [36,37]. EMT describes the physiologic and pathologic process by which epithelial cells de-differentiate into mesenchymal cells. Epithelial cells, which are normally polarized with intact cell-to-cell junctions, de-differentiate into mesenchymal cells which allows for wound healing in normal cells, but also migration and metastatic spread in tumor cells [38]. Changes in morphology and signaling lead to conversion to a poorly differentiated cell [36,38]. Epithelial cells require the structural stability of adherens junctions which are comprised of cadherin proteins [39]. E-cadherin is a calcium dependent transmembrane glycoprotein that facilities extracellular interactions with other epithelial cells [39]. The downregulation of E-cadherin has been noted to be a hallmark of early stages of EMT [40,41,42]. The emergence of EMT related transcription factors (EMT-TF), such as TWIST1 and SNAIL, silence E-cadherin expression through direct binding to the E-cadherin gene, which disrupts cell junctions and allows for tumor migration [43]. 

Research has shown that anti-androgen treatments such as enzalutamide have resulted in the upregulation of EMT-TF, such as TWIST1 and SNAIL, via the Twist1/Androgen Receptor (AR) axis [44]. Patients on ADT with high TWIST1 expression may benefit from TWIST1 inhibition to prevent EMT [45]. TWIST1 inhibitors have been studied in lung cancer, and results have shown cell growth inhibition and apoptosis [46,47]. Martin et al., proposed EMT as a mechanism of resistance to Cabazitaxel and antiandrogen therapy in advanced prostate cancer, thus justifying more research inquiry into the pathway [48].

Dysregulated ABI1, a protein involved in cellular cytoskeleton stabilization and signaling, may also contribute to the EMT process [49]. Downregulation of ABI1 is associated with loss of E-cadherin, the key protein involved in maintaining the adherens junction [49]. This may propel disease progression and metastatic spread of tumor through activation of EMT. ABI1 loss has also been associated with upregulated STAT3 activity [49]. STAT3 is a master regulator of EMT transcriptional programming that promotes cellular adhesion, migration, proliferation and differentiation [38,50,51]. Gujral et al. identified the critical mechanism of STAT3-mediated invasion through activation of noncanonical WNT pathway [52]. WNT pathway is one of the key pathways associated with prostate tumor progression and invasion [53,54,55]. 

## 5. Targeting STAT3 as Master Regulator in nmCRPC

Much has been said about inhibiting STAT3 in cancer. STAT3 is an established target in the majority of advanced human tumors including prostate cancers [56,57]. STAT3 is elevated in prostate cancer cells as well as in many types of tumor-infiltrating immune cells, therefore pharmacological approaches aim to downregulate STAT3 function. Another justification for potentially targeting STAT3 in nonmetastatic CRPC is the fact that inhibition of STAT3 might prevent treatment-induced neuroendocrine-like prostate tumor phenotype, also termed as t-NEPC. Incidence of these type of tumors is expected to rise upon increased use of novel anti-AR agents [29,58,59]. These tumors alike classical NEPC tumors [59] are likely to be challenging to treat as they are resistant to anti-AR agents and are usually treated with platinum therapy subsequent to exhausting taxane therapy [56,59,60,61].

The direct effect of STAT3 targeting depends on the genetic background of cancer cells. In PTEN-deficient cancer cells, STAT3 may act as a tumor suppressor [62] and promote tumor senescence by transcriptional regulation of ARF-p21-P53 axis [63]. The disruption of STAT3 signaling in PTEN null prostate cancer cells can promote tumor growth in mice [63]. However, targeting STAT3 in the whole tumor microenvironment, including tumor-associated myeloid cells, was shown to generate potent antitumor effects independently from PTEN status of cancer cells [57]. These preclinical results underscore therapeutic potential and priority in targeting STAT3 activity in tumor-associated immune cells rather than in cancer cells alone [57,64]. Synergistic activity of anti-STAT3 inhibitors on tumor microenvironment might be more important than their direct cytotoxic effect on prostate cancer cells [65]. STAT3 is commonly activated in immunosuppressive myeloid cells such as macrophages and MDSCs [66,67]. Hence, targeting STAT3 in the tumor microenvironment allows for tumor shrinkage due to proper immune system re-activation [57,64].

**Figure 2:**
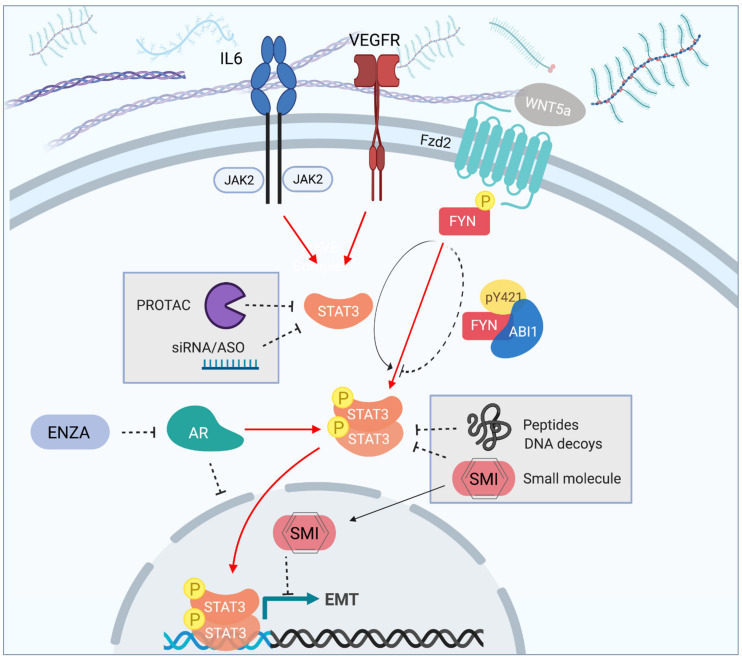
STAT3 as potential target pathway in nmCRPC. Major types and mechanisms of current STAT3 inhibitors: siRNA/ASO, polypeptides, DNA decoys, small molecule and/or PROTAC inhibitors. The goal of inhibitors is to decrease levels of STAT3 mRNA or protein (siRNA/ASO or PROTAC), to inhibit STAT3 nuclear translocation (DNA decoys), or the transcriptional activity by interfering with DNA binding (peptides or small molecule inhibitors). Major pathways regulating STAT3 signaling in prostate epithelial cells are depicted.

Multiple approaches to inhibit STAT3 levels or activity have been tested in preclinical studies (Figure 2) [68]. Small molecule Janus kinase inhibitors allow for targeting upstream regulators of STAT3 activity [69,70]. Peptide, decoy DNA or small molecule-based approaches aim at targeting the SH2 domain of STAT3 to prevent dimerization, which is involved in DNA binding activity and STAT3 transcriptional activity [69,70]. Efficient downregulation of STAT3 can be obtained by either oligonucleotide-based inhibitors, such as antisense oligonucleotides [57], or, by small molecules acting as proteolysis targeting chimeras (PROTACs) [71].

ABI1 regulates STAT3 expression through SRC kinase FYN, providing a potential novel strategy for STAT3 targeting [49]. Enhanced STAT3 levels are associated with enzalutamide resistance [72]. Therefore, loss or downregulation of ABI1 with overactive STAT3 signaling [49] may contribute to enzalutamide resistance. This suggests a potential role of ABI1 as a novel biomarker for early EMT events, as well as STAT3- mediated enzalutamide resistance. This also suggests a potential role for STAT3- inhibitors to resensitize tumors to enzalutamide as well as other ADTs [72]. 

## 6. Conclusions

Given that 50% of men with nmCRPC will undergo progression to mCRPC, novel therapies are needed. Currently, three FDA approved anti-androgen therapies are used in this sphere: apalutamide, enzalutamide, and darolutamide. Numerous targets for non-androgen pathways have been explored previously. This review introduces the novel concept of targeting the EMT process early on in nonmetastatic prostate cancer, as EMT plays a key role in disease progression and may serve as a potential target for future therapeutics and biomarkers. Further exploration of disrupted signaling pathways and cellular architecture may uncover potential opportunities for disease control. 

## Data Availability

All data reviewed in the paper is either publicly available and/or previously published.
